# Non-Syndromic or Anomaly-Associated Genes (MYH3, GREM1, IRF6) and Their Proteins in Unilateral Right Cleft Tissue

**DOI:** 10.3390/ijms27104158

**Published:** 2026-05-07

**Authors:** Alise Elizabete Rone, Anna Junga, Ilze Akota, Mara Pilmane

**Affiliations:** 1 Institute of Anatomy and Anthropology, Riga Stradins University, 16 Dzirciema Street, LV-1007 Riga, Latvia; anna.junga@rsu.lv (A.J.); mara.pilmane@rsu.lv (M.P.); 2 Department of Oral and Maxillofacial Surgery, Riga Stradins University, 16 Dzirciema Street, LV-1007 Riga, Latvia; ilze.akota@rsu.lv; 3 Cleft Lip and Palate Centre, Institute of Stomatology, Riga Stradins University, 20 Dzirciema Street, LV-1007 Riga, Latvia

**Keywords:** MYH3, IRF6, GREM1, cleft lip, IHC, CISH

## Abstract

Human unilateral cleft lip morphopathogenesis is a complex process involving multiple genes and proteins. Factors such as myosin heavy chain 3 (*MYH3*), interferon regulatory factor 6 (*IRF6*), and Gremlin 1 (*GREM1*) are implicated in craniofacial development; however, their precise role in unilateral cleft formation remains unclear, limiting improvements in treatment strategies. Immunohistochemistry (IHC) for MYH3, IRF6, and GREM1 proteins and chromogenic in situ hybridization (CISH) for *IRF6* and *GREM1* genes were used to analyze postnatal unilateral right cleft lip tissue (ten patients) and control tissue (six patients). The semi-quantitative counting method was applied, followed by statistical analysis. IHC revealed increased MYH3 expression in cleft muscle tissue and elevated IRF6 expression in the epithelium, whereas GREM1 showed low expression, with significant differences in connective tissue. *CISH* demonstrated increased *IRF6* gene expression in the cleft epithelium, whereas *GREM1* expression did not differ from controls. Multiple statistically significant correlations were identified, highlighting their potential involvement in cleft morphopathogenesis.

## 1. Introduction

Non-syndromic cleft lip and/or palate is one of the most common congenital craniofacial anomalies, arising from impaired growth, differentiation, and fusion of facial prominences during early embryonic development [1,2,3]. Unlike syndromic clefts, non-syndromic clefts occur in isolation and are considered a multifactorial condition involving complex interactions between multiple genes, regulatory pathways, and environmental factors [4]. Despite considerable progress in craniofacial genetics, the molecular mechanisms underlying clefts—particularly those contributing to phenotypic variability and laterality—remain incompletely understood.

Epidemiological studies consistently show that unilateral clefts are more common than bilateral forms, and that left-sided clefts occur more frequently than right-sided clefts across diverse populations [1,2,5]. However, the biological basis of this laterality bias remains incompletely understood. The biological basis of this asymmetry is not fully explained; however, increasing evidence suggests that localized disturbances in gene and protein presence within cleft-affected tissues may play a significant role [6]. Therefore, investigation of molecular alterations directly in human cleft tissue has become an important strategy for elucidating disease mechanisms.

Interferon Regulatory Factor 6 (*IRF6*) is a key regulatory gene involved in craniofacial development and epithelial differentiation. It plays a critical role in periderm formation and in the regulation of epithelial seam disintegration during lip and palate fusion, processes essential for normal orofacial morphogenesis [7,8]. Genetic studies across diverse populations have consistently demonstrated that variants in *IRF6* are strongly associated with non-syndromic cleft lip with or without cleft palate, supporting its role as one of the most robust and replicated susceptibility loci for orofacial clefts [5,9,10]. These findings highlight *IRF6* as a major genetic contributor to cleft risk within a multifactorial etiological framework.

Functional studies further indicate that reduced or disrupted *IRF6* activity impairs epithelial differentiation and compromises epithelial integrity during craniofacial development, leading to abnormal persistence of epithelial structures and failure of proper fusion processes [7,11]. At the tissue level, previous studies, including those by Pilmane et al., have demonstrated altered *IRF6* expression in human cleft lip tissues compared with controls, suggesting that dysregulation of this pathway is detectable not only at the genetic level but also within affected craniofacial tissues [12,13].

Taken together, these findings support a model in which IRF6 contributes to cleft lip pathogenesis through coordinated effects on epithelial differentiation and tissue remodeling, acting within a broader network of genetic and signaling pathways rather than as a sole determinant of disease.

*GREM1* (Gremlin 1) encodes a secreted antagonist of bone morphogenetic protein (*BMP*) signaling, a pathway essential for craniofacial patterning, cell proliferation, and morphogen gradient regulation [14,15]. Genome-wide association and linkage studies have identified loci near *GREM1* as risk factors for clefts [16,17]. Experimental models demonstrate that altered Gremlin–BMP interactions can result in abnormal craniofacial development [18]. However, despite its biological relevance, GREM1 gene and protein presence in human unilateral cleft tissue remains poorly characterized, representing an important gap in current knowledge.

Variants at the *GREM1* locus have been associated with susceptibility to non-syndromic cleft lip with or without cleft palate in multiple populations, and genome-wide studies have identified this region as a significant risk locus, supporting a contributory role of *GREM1* in craniofacial development [19,20].

Myosin heavy chain 3 (*MYH3*), encoding embryonic myosin heavy chain, is primarily known for its role in muscle development and congenital contracture syndromes [21]. Increasing evidence suggests that embryonic myosins contribute to tissue organization, cellular migration, and biomechanical forces during craniofacial morphogenesis [22]. Animal studies have demonstrated that *MYH3* dysfunction can be associated with cleft palate formation, indicating a potential role beyond muscle differentiation [23]. Nevertheless, *MYH3* expression in non-syndromic cleft-affected human tissue has received minimal investigation.

Building upon the methodological framework, the present study focuses on *MYH3*, *GREM1*, and *IRF6* gene and protein presence in unilateral right non-syndromic cleft tissue. Analysis of these markers may provide insight into localized molecular disturbances and contribute to improved molecular characterization of cleft lip tissue.

This study will mainly focus on assessing the presence of *MYH3*, *GREM1* and *IRF6* proteins and by assessing the same factor gene-signal-containing cells in right cleft affected tissue and control tissue.

## 2. Results

### 2.1. Immunohistochemistry (IMH)

Immunohistochemical analysis indicated the presence of MYH3, GREM1 and IRF6 in all patient and control group specimens, with a variable distribution of immunoreactive cells.

**Figure 1:**
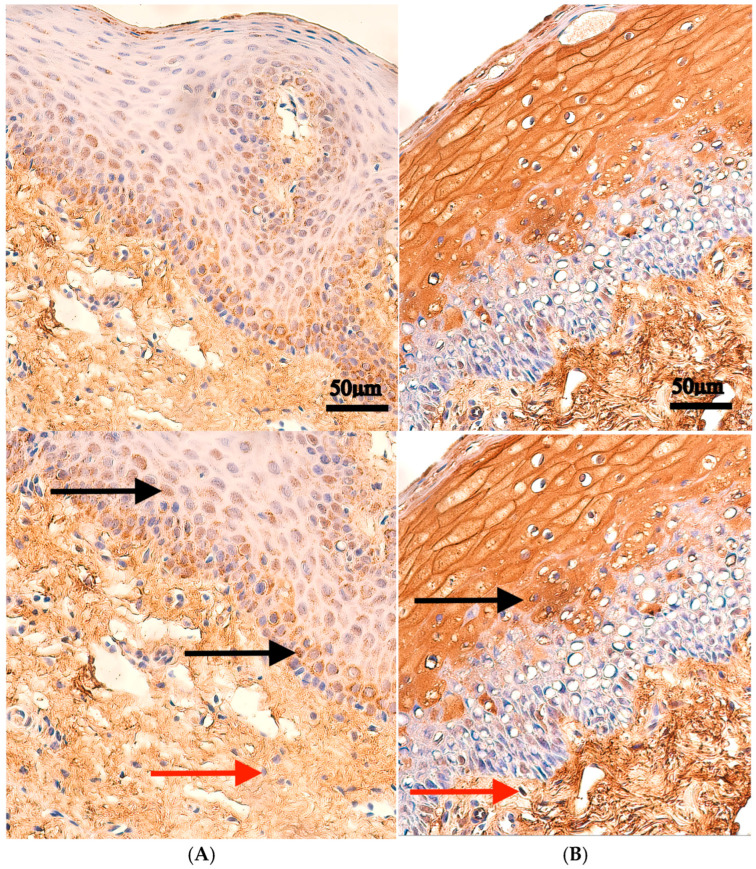
Myosin 3 immunohistochemistry in control and cleft lip tissue. Upper panels are shown at original magnification (×200), while lower panels are enlarged for improved visualization of staining. (**A**) Control tissue with few positive Myosin 3-containing epithelial cells (black arrows), and occasionally Myosin 3-positive cells within connective tissue (red arrows), ×200. (**B**) Cleft tissue with a moderate number of Myosin 3-containing epithelial cells (black arrow), and a few positive Myosin 3-positive cells within connective tissue (red arrow), ×200.

Myosin 3 protein was found in every individual in the patient group with a variable distribution. The median value of Myosin 3-positive cells in the patient tissue epithelium was a few (+) positive structures, but the connective tissue and endothelium both had median values of occasionally (0/+) positive structures, and muscle tissue had a moderate to numerous amount (++/+++) of positive structures. In the control group tissue, the median value was a few (+) positive structures in the epithelium, occasionally (0/+) positive structures in the connective tissue, and no positive structures in both the endothelium and muscle tissue (Table 1, Figure 1).

Statistically significant correlations were observed between controls and the patient group in the number of positive structures in the muscle tissue (*p* = 0.003), but not in the epithelium (*p* = 0.118), connective tissue (*p* = 0.313) or endothelium (*p* = 0.093) (Table 2).

**Figure 2:**
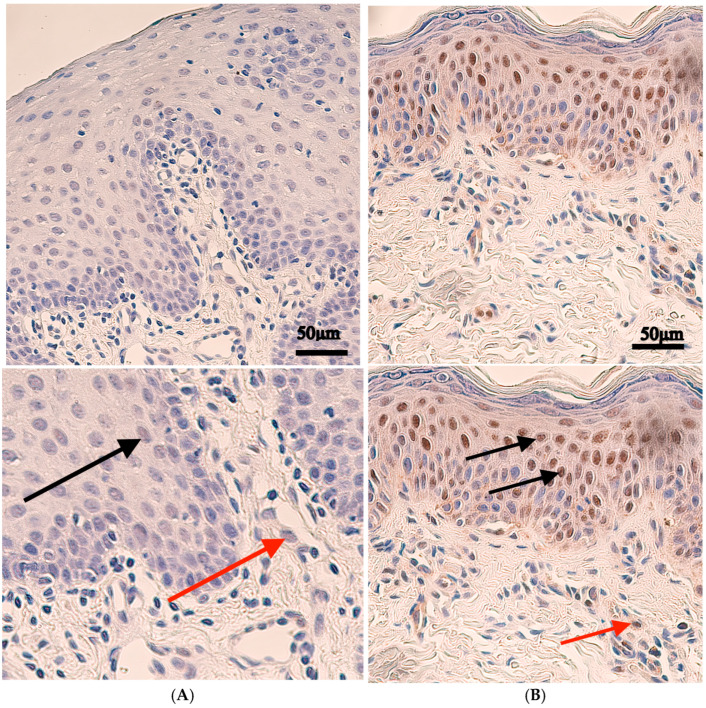
IRF6 immunohistochemistry in control and cleft lip tissue. Upper panels are shown at original magnification (×200), while lower panels are enlarged for improved visualization of staining. (**A**) Control tissue with no positive IRF6 epithelial cells (black arrow), and no positive IRF6 cells within connective tissue (red arrow), and no IRF6-positive endothelial cells, ×200. (**B**) Cleft tissue with moderate number of IRF6-positive epithelial cells (black arrow); occasionally IRF6-positive cells within connective tissue (red arrow), ×200.

IRF6 protein-positive cells were also identified in every patient’s tissue with variable distribution. The median number of positive cells in the epithelium was from a few to moderate number (+/++) of positive structures, and there was a similar median expression of occasionally (0/+) positive structures in the connective tissue, endothelium and muscle tissue. The control tissue revealed similar expression with occasionally (0/+) positive structures in both the epithelium and connective tissue and no positive structures in the endothelium and muscle tissue (Table 1, Figure 2).

Statistically significant differences were observed between controls and patients with IRF6 immunoreactive epithelium (*p* = 0.003) and an almost statistically significant difference was found in the *IRF6*-endothelium (*p* = 0.056), but no significant changes were found between connective tissue (*p* = 0.635) and muscle tissue (*p* = 0.428) (Table 2).

**Figure 3:**
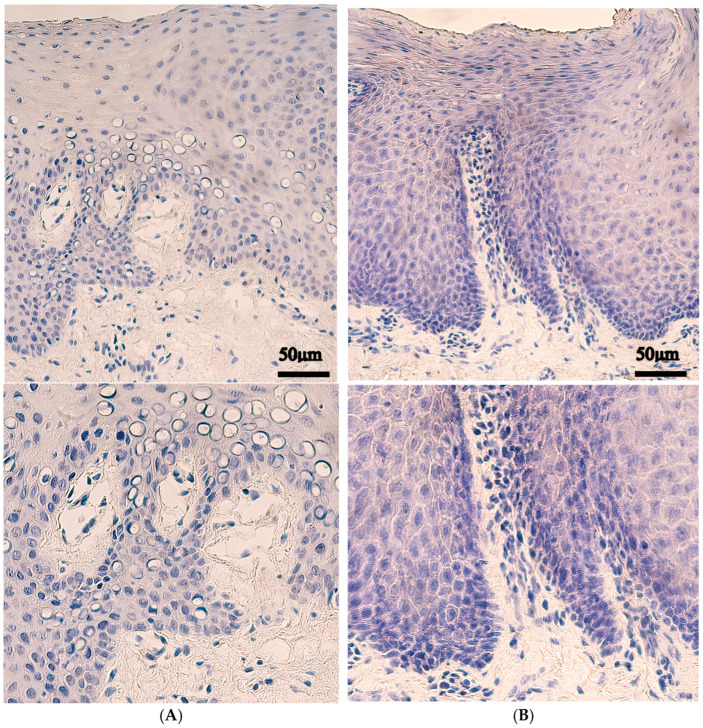
Gremlin immunohistochemistry in control and cleft lip tissue. Upper panels are shown at original magnification (×200), while lower panels are enlarged for improved visualization of staining. (**A**) Control tissue with no Gremlin-positive epithelial cells, no cells within connective tissue and muscle cells and no Gremlin-positive endothelial cells, ×200. (**B**) Cleft tissue with no Gremlin-positive epithelial cells, positive cells within connective tissue, endothelial cells or muscle tissue cells, ×200.

Gremlin was much less expressed with a median value of occasionally (0/+) positive structures in the epithelium and no positive structures in the connective tissue, endothelium and muscle tissue. The control tissue revealed similar median expression levels with occasionally (0/+) positive cells in the epithelium and connective tissue and no positive structures in both the endothelium and muscle tissue (Table 1, Figure 3).

Statistically significant changes were found between control and patients with Gremlin-positive connective tissue (*p* < 0.001), but no statistically significant changes were found in the epithelium (*p* = 0.635), endothelium (*p* = 0.875) or muscle tissue (*p* = 0.792) (Table 2).

### 2.2. Chromogenic In Situ Hybridization (CISH)

*IRF6* and *GREM1* gene-signal-containing cells were identified in both the control and patient groups.

**Figure 4:**
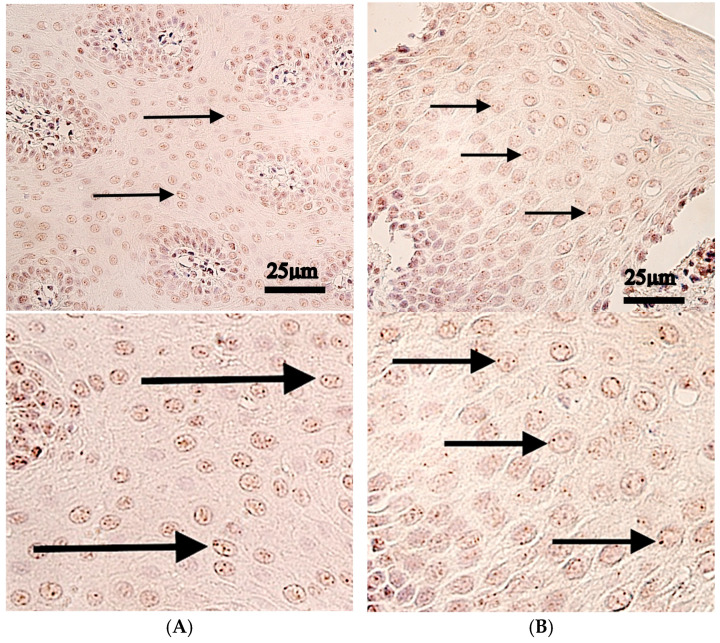
*IRF6* chromogenic in situ hybridization in control and cleft lip tissue. Upper panels are shown at original magnification (×400), while lower panels are enlarged for improved visualization of staining. (**A**) Control tissue with moderate IRF6-positive epithelial cells (black arrows), ×400. (**B**) Cleft tissue with a moderate number of IRF6-positive epithelial cells (black arrows), and no IRF6-positive cells within connective tissue, muscle cells or endothelial cells, ×400.

The median values of *IRF6* genes in the patient tissue were as follows: few to moderate (+/++) number of positive structures in epithelium, occasionally (0/+) positive structures in both connective tissues, no positive structures in muscle tissue. The control tissue epithelium and control tissue exhibited median value of occasionally (0/+) positive structures, but in the endothelium and muscle tissue no positive structures were found (Table 3, Figure 4).

Statistically significant changes were found between controls and patients with Gremlin-positive structures in the epithelium (*p* = 0.031), but not in the connective tissue (*p* = 0.263), endothelium (*p* = 0.118), or muscle tissue (*p* = 1.000) (Table 2).

**Figure 5:**
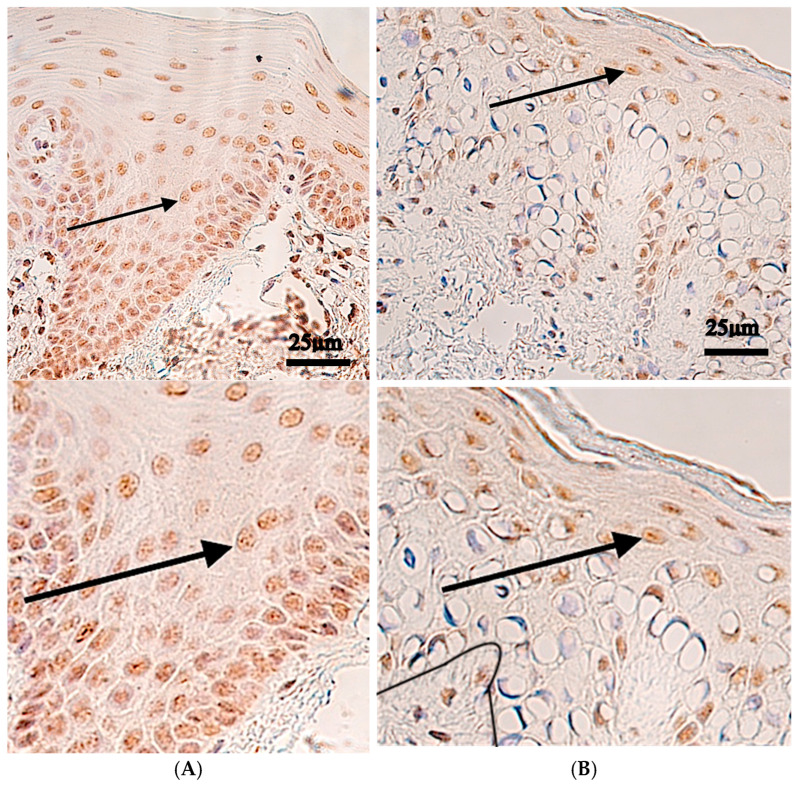
*GREM1* chromogenic in situ hybridization in control and cleft lip tissue. Upper panels are shown at original magnification (×400), while lower panels are enlarged for improved visualization of staining. (**A**) Control tissue with occasionally Gremlin-positive epithelial cells (black arrow), no positive cells within connective tissue, muscle cells and no Gremlin-positive endothelial cells, ×400. (**B**) Cleft tissue with occasionally Gremlin-positive epithelial cells (black arrow), and no Gremlin-positive cells within connective tissue, muscle cells or endothelial cells, ×400.

*GREM1* gene signals had low expression levels with occasionally (0/+) positive structures in the epithelium and connective tissue, and no positive structures in the endothelium and muscle tissue. An identical median value was found in the control tissue with occasionally (0/+) positive structures in the epithelium and connective tissue and no positive structures in the endothelium and muscle tissue (Table 3, Figure 5).

Statistically significant changes were not identified between control and patient Gremlin-positive tissues in neither the epithelium (*p* = 0.118), nor connective tissue (*p* = 0.958), nor endothelium (*p* = 1.000), nor muscle tissue (*p* = 0.875) (Table 2).

### 2.3. Statistical Analysis

Statistically significant differences were observed between *MYH3*-positive cells in the muscle tissue of patients and the control (U = 4, *p* = 0.003), *IRF6*-positive cells in the endothelium of patients and the control (U = 4, *p* = 0.003), *GREM1*-positive cells in the connective tissue of patients and the control (U = 2, *p* < 0.001), and *IRF6* gene-signal-containing cells in the epithelium of patients and the control (U = 10.5, *p* = 0.031). An almost statistically significant difference was observed between *IRF6*-positive cells in the endothelium of patients and the control (U = 12, *p* = 0.056) (Table 2).

#### 2.3.1. Correlations in Patient Group

The patient group had four statistically significant correlations of which all were positive correlations (one very strong, three strong).

The strongest correlation was found between *IRF6*-positive cells in muscle tissue and *MYH3*-positive cells in muscle tissue (Rs = 0.826; *p* = 0.003). Strong correlations were found between *GREM1*-positive cells in the endothelium and *MYH3*-positive cells in connective tissue (Rs = 0.667; *p* = 0.035), *GREM1*-positive cells in muscle tissue and *MYH3*-positive cells in connective tissue (Rs = 0.764; *p* = 0.010) and *IRF6*-positive cells in the endothelium and *IRF6* gene-signal-containing cells in the endothelium (Rs = 0.746; *p* = 0.013) (Table 4). No statistically significant negative correlations were found.

#### 2.3.2. Correlations in Control Group

The control group had two statistically significant correlations—none were positive correlations, and two were negative (two very strong). Very strong significant negative correlations were found between Gremlin-positive cells in connective tissue and *MYH3*-positive cells in the epithelium (Rs = −0.980; *p* < 0.001) and *MYH3* positive cells in the endothelium and *GREM1* gene-containing-cells in connective tissue (Rs = −0.980; *p* < 0.001) Table 4.

## 3. Discussion

Our study demonstrates a significant presence of *MYH3*-positive cells in cleft-affected muscle tissue, as identified by immunohistochemistry, as well as altered *IRF6* expression in the epithelium, detected by both IHC and CISH. However, the observed subcellular distribution should be interpreted with caution due to limitations related to antibody specificity.

In contrast, immunoreactivity in the connective tissue and endothelium did not differ substantially between patients and controls for any of the investigated factors—*MYH3*, *IRF6*, or *GREM1*. These findings suggest that alterations in *MYH3* and *IRF6* expression in cleft-affected tissues may be associated with cleft pathogenesis; however, the underlying molecular mechanisms remain to be clarified.

### 3.1. MYH3 in Muscle Tissue

Our observation of elevated *MYH3* expression in cleft-affected muscle tissue is consistent with previous studies linking *MYH3* to craniofacial development. A pivotal study in Limousin cattle demonstrated a relationship between *MYH3* dysregulation and cleft defects, where animals harboring a recessive *MYH3* mutation exhibited markedly reduced mRNA expression and an absence of detectable *MYH3* protein in muscle tissues, particularly in extraocular and craniofacial muscles [24]. The mutation resulted in premature termination of the *MYH3* protein, likely leading to nonsense-mediated mRNA decay and reduced protein levels. Although this model reflects reduced *MYH3* function, it suggests that precise regulation of *MYH3* expression is critical for normal craniofacial development. Both insufficient and excessive expression may therefore reflect disrupted muscle differentiation or contractile function, potentially affecting coordinated orofacial morphogenesis.

The increased MYH3 signal observed in cleft tissue should be interpreted with caution. While this finding may suggest enhanced MYH3 expression, it may alternatively reflect a higher proportion of muscle cells within the analyzed regions. Given that the present study was designed to examine the spatial distribution of selected markers using immunohistochemistry and CISH, rather than to quantitatively assess gene or protein expression or perform detailed cell-type characterization, it was not possible to discriminate between these possibilities. The inclusion of additional muscle-specific markers would be required to distinguish between changes in cellular composition and true upregulation at the single-cell level. Therefore, the observed differences are best interpreted as alterations in signal distribution within the tissue architecture. Future studies integrating multiple muscle cell markers and quantitative approaches will be necessary to further elucidate the role of MYH3 in the context of cleft lip pathology.

Although *MYH3* is not expressed in the palate itself, its expression in craniofacial muscles during embryogenesis is important for normal orofacial development. Palatal shelf elevation and fusion depend on coordinated movements of the tongue and surrounding musculature; therefore, altered *MYH3* expression may be associated with changes in muscle dynamics that could indirectly influence palatal shelf positioning.

Supporting this concept, a recent proteomic study in animal models of cleft palate identified *MYH3* as a hub protein exhibiting differential expression in cleft conditions, along with other motor proteins such as myosin heavy chain 8 (*MYH8*) and myosin heavy chain 11 (*MYH11*) [25]. These findings support the idea that dysregulation of muscle-associated contractile proteins may be linked to cleft phenotypes.

In humans, direct causal evidence linking *MYH3* expression changes to cleft lip remains limited, as most studies focus on other *MYH* family members or cleft palate rather than cleft lip specifically. However, *MYH3*-associated disorders, such as autosomal dominant spondylocarpotarsal synostosis, indicate that MYH3 is involved in postnatal muscle and skeletal development, including craniofacial structures, and that mutations may influence transforming growth factor beta (TGF-β) signaling [26]. Notably, some individuals with *MYH3* mutations present with cleft palate, suggesting that craniofacial anomalies may be part of the broader *MYH3*-associated phenotype. Collectively, these findings support an association between *MYH3* dysregulation and cleft-related developmental disturbances, although a direct mechanistic link remains to be established.

### 3.2. IRF6 Expression in Epithelium

*IRF6* is a well-established regulator of epithelial differentiation and fusion during lip and palate development. Smane and Pilmane (2016) reported reduced *IRF6* immunoreactivity in oral epithelial tissues from children with complete bilateral non-syndromic cleft lip and palate compared with controls, suggesting an altered epithelial differentiation capacity [12]. This supports the clinical relevance of *IRF6* dysregulation in cleft pathology.

IRF6 is expressed in oral epithelium and at fusion sites, including the medial edge epithelium (MEE), where epithelial seam breakdown is required for palatal fusion [26]. Although human studies remain limited, animal models have established a central role for IRF6 in craniofacial morphogenesis. In mice, *IRF6* is expressed in oral/ectodermal epithelium and MEE prior to and during palatal fusion, and mutations in *IRF6* result in impaired epithelial differentiation and abnormal tissue adhesions, both associated with clefting [8].

IRF6 expression is regulated by p63 and is essential for keratinocyte differentiation during palate development; disruption of this regulatory pathway contributes to cleft phenotypes [27]. Additionally, experimental murine studies indicate that IRF6 participates in epithelial–mesenchymal transition (EMT) via *SNAI2*, a process required for epithelial seam breakdown during palatal closure [28]. Together, these findings support the importance of IRF6-mediated epithelial regulation in craniofacial development.

Further human evidence suggests that *IRF6* expression is influenced by genetic variation. In a Deutero-Malay population, individuals carrying the *IRF6* rs2235371 heterozygous risk genotype exhibited increased IRF6 mRNA expression in nasal and oral epithelium compared with GG homozygotes [29]. Collectively, these data indicate that IRF6 is involved in cleft-related epithelial processes and that its expression is modulated by both genetic and developmental factors. However, we are not indicating functional genetic alterations or gain-of-function effects, and we acknowledge that future studies integrating genomic sequencing would be required to explore this hypothesis.

### 3.3. Gremlin Expression

Gremlin, a bone morphogenetic protein (*BMP*) signaling antagonist, was detected at low levels in both patient and control tissues, with occasional positive staining in the epithelium and connective tissue, and minimal expression in the endothelium and muscle. A statistically significant difference between patients and controls was observed only in connective tissue (*p* < 0.001). Overall, the low expression levels and limited tissue-specific differences suggest that Gremlin is not a predominant factor in human cleft lip morphogenesis based on the present data.

BMP signaling is essential for craniofacial development, regulating cell proliferation, differentiation, and apoptosis during facial morphogenesis. Its activity is tightly controlled by extracellular antagonists such as Gremlin, Noggin, and Chordin [30,31,32]. Experimental animal studies have shown that disruption of BMP antagonists, including Gremlin, can lead to craniofacial abnormalities through altered epithelial–mesenchymal interactions [33].

In humans, however, the role of *GREM1* appears to be more complex. Previous genetic studies have reported associations between variants in *GREM1* and non-syndromic orofacial clefts, suggesting that it may contribute to susceptibility within a broader genetic context [19]. These findings indicate that, despite the low expression observed in the present study, Gremlin cannot be excluded as a contributory factor in cleft lip development.

In the present study, Gremlin expression in human cleft lip tissue was generally minimal and comparable to controls, suggesting that its contribution, if any, is likely indirect. The isolated difference observed in connective tissue may reflect subtle changes in extracellular matrix signaling or mesenchymal activity rather than a primary pathogenic mechanism.

Connective tissue plays an important role in epithelial–mesenchymal crosstalk during lip and palate formation, and even small alterations in signaling regulators may influence local morphogenesis without producing widespread changes in expression [30,31,34]. The lack of robust Gremlin expression across tissue compartments contrasts with experimental models, indicating species-specific differences in BMP signaling regulation and compensatory mechanisms involving other BMP antagonists [35].

The role of GREM1 in cleft lip pathogenesis remains to be interpreted with caution. Although the present findings do not support a prominent or consistent alteration in GREM1 signal distribution in cleft tissues, the limited sample size and the semi-quantitative nature of the analyses preclude definitive conclusions regarding its functional relevance. Importantly, previous genetic studies have identified associations between GREM1 variants and non-syndromic orofacial clefts, suggesting that this factor may contribute to craniofacial development in a context-dependent manner [19,20]. These observations indicate that GREM1 may not act as a primary driver of cleft lip formation but could instead participate as one component within a complex regulatory network involving multiple signaling pathways, genetic susceptibility factors, and environmental influences. Therefore, further investigations integrating quantitative expression analyses, larger patient cohorts, and functional studies are required to clarify the precise contribution of GREM1 to cleft lip etiology.

### 3.4. Study Limitations

Several limitations should be acknowledged. The study relied on IHC and CISH to assess relative protein and gene expression; inclusion of quantitative approaches such as ELISA could provide more precise concentration characterization. However, our findings reflect changes in signal distribution within the tissue architecture rather than definitive evidence of differential expression per cell.

Additionally, the modest sample size may limit statistical power and generalizability, while ethical constraints restricted access to pediatric control tissues. Age differences between groups may represent a potential confounder, although sampling during the primary dentition period partially mitigates this effect. A limitation of the present study is the reliance on commercially available antibodies without additional independent validation. Although manufacturer-provided data were considered, some of the antibodies used are not extensively characterized in the literature. Furthermore, the observed subcellular localization patterns—particularly for *MYH3*, which is typically described as a cytoplasmic protein—should be interpreted with caution.

Despite these limitations, the use of human tissue represents a key strength, as most cleft research relies on animal models whose interspecies differences may limit direct translational relevance to human craniofacial development. Future investigations incorporating connective tissue, epithelial signaling, and myogenic factors will be necessary to further improve understanding of lip development and associated pathological conditions.

## 4. Materials and Methods

### 4.1. Ethics Approval

Tissue material was obtained from patients and controls in the Cleft, Lip and Palate Centre of the Institute of Stomatology of Riga Stradins University, after all parents of the patients were fully informed about the nature of this study and had signed agreements for voluntary donation. Tissue morphological analysis was conducted in the RSU Institute of Anatomy and Anthropology. This research was conducted in accordance with the 1975 Helsinki Declaration (as revised in 2008). The study was independently reviewed and approved by the Ethical Committee of the Riga Stradins University with two following permissions—1st on 22 May 2003; 2nd on 15th December 2025 (No. 2-PEK-4/1372/2025).

The ethical committee approval Nr. 2-PEK-4/595/2022 for the use of the control group’s tissues was issued on 14 December 2022.

### 4.2. Selection Criteria of Patient Tissue Samples

Patients were selected according to the following inclusion criteria:

- Diagnosis of right side unilateral cleft lip and palate (cheilognathouranoschisis dextra);
- Surgery performed at an age before/during primary dentition;
- Absence of other congenital anomalies;
- Absence of malignancy, active or acute inflammation or any other changes in the tissue;
- Age during primary dentition without presence of permanent dentition.

The exclusion criteria were defined as follows:

- Coexistence of additional congenital anomalies;
- Evidence of active or acute inflammatory conditions;
- Medical conditions that represent contraindications to cleft repair surgery;
- Patients in the mixed dentition phase (6–12 years of age) or those with fully established permanent dentition;
- Incomplete clinical records or lack of informed consent.

### 4.3. Description of Selected Patients

In total, 10 patients were selected, and from these patients, 10 samples of lip tissue were obtained during cheiloplastic surgery in the Cleft Lip and Palate Centre of the Institute of Stomatology of Riga Stradins University. The patient age varied from three months to 18 months. Six of the patients selected were male, and four were female. One of the patients had a family history of clefts and a mother with hepatitis B; two had mothers with history of infection during pregnancy; and seven patients had parents with no previous history of illnesses. All patients underwent cheiloplastic surgery. Information about each patient’s age, sex, and family anamnesis is summarized in Table 5.

### 4.4. Selection Criteria for Control Tissue Samples and Control Group

Control samples were selected according to the following criteria:

- Patients had no craniofacial cleft upon examination, and no family history;
- Absence of secondary pathologies and congenital anomalies;
- No visible damage or inflammation of oral cavity.

For the control group, in total, six tissue samples were acquired from the Institute of Anatomy and Anthropology of Riga Stradins University during post-mortem necropsies. Four of the selected controls were female, and two were male. The control age varied from newborn to 24 weeks old. For one of the controls, the cause of death was birth asphyxia; for one, it was sudden infant death syndrome; and for one, it was abortion due to maternal health status; for the remaining three, material was taken during frenectomy Table 6.

### 4.5. Immunohistochemistry (IHC) Analysis

Immunohistochemical evaluation was used to determine the number of *MYH3*-, *IRF6*- and *GREM1*-positive cells in primary dentition study group and control group using a conventional streptavidin–biotin detection method, a widely applied approach for antigen visualization in formalin-fixed paraffin-embedded tissues [33]. IHC based on technical specifications set by manufacturers was performed with the following antibodies: *IRF6* (orb559090, 1:100, Biorbyt LLC., Durham, NC, 27709, USA), *Myosin-3* (orb185150, 1:200, Biorbyt LLC., Durham, NC, 27709, USA), *GREM1* (orb500806, 1:200, Biorbyt LLC., Durham, NC, 27709, USA). Primary antibodies were diluted in antibody diluent solution (code 938B-05, Cell Marque™, Rocklin, CA, USA) according to the manufacturer’s recommendations.

Prior to antibody incubation, tissue sections underwent standardized preparation procedures [36]. Paraffin-embedded samples were deparaffinized and rehydrated through graded alcohol solutions followed by rinsing in distilled water. Sections were washed twice in Tris buffer solution for 5 min each. Antigen retrieval was performed by heat-induced epitope retrieval using boiling EDTA buffer in a microwave oven for 20 min, followed by gradual cooling, a procedure known to enhance antigen exposure and antibody binding efficiency [37]. After retrieval, slides were washed again in Tris buffer (2 × 5 min). Endogenous peroxidase activity was blocked with 3% hydrogen peroxide for 10 min to prevent nonspecific background staining, followed by additional washing in Tris buffer.

Immunodetection was performed through sequential incubation steps. Tissue sections were incubated with primary antibodies for 1 h at room temperature and subsequently washed three times with Tris buffer. Slides were then exposed to the HiDef Detection™ reaction amplifier (code 954D-31, Cell Marque™, Rocklin, CA, USA) for 10 min at room temperature, followed by three additional washing steps of 5 min each in Tris buffer.

For visualization, sections were treated with DAB+ chromogen using the DAB Substrate Kit (code 957D-60, Cell Marque™, Rocklin, CA, USA) for 10 min, allowing enzymatic conversion into a brown reaction product at antigen sites [33]. Slides were rinsed under running water and counterstained with Mayer’s hematoxylin (code 05-M06002, Bio Optica Milano S.p.A., Milan, Italy). Subsequently, tissues were dehydrated through increasing ethanol concentrations (70–90%), cleared using carboxylic acid and xylene, and mounted with coverslips.

To minimize observational bias, all samples were coded prior to evaluation. Patient and control specimens were assigned pseudonymized numerical identifiers corresponding to each investigated marker. Slide assessment was performed under blinded conditions, after which the coding was decoded and samples were categorized into patient and control groups for further analysis, consistent with recommended practices for reducing assessment bias in histopathological studies [38].

### 4.6. Chromogenic In Situ Hybridization (CISH) Analysis

Chromogenic in situ hybridization was performed using a standardized protocol designed for chromogenic detection of nucleic acid targets in formalin-fixed, paraffin-embedded tissue sections, following contemporary in situ hybridization methodological principles [37,39]. The ZytoDot2C CISH Implementation Kit (ZytoVision GmbH, Bremerhaven, Germany) was used with the following probes: *IRF6* probe (IRF6-20-D-R, Empire Genomics Corp., Williamsville, NY, USA); *GREM1* probe (GREM1-20-D-R, Empire Genomics Corp., Williamsville, NY, USA).

Prior to probe application, slides were subjected to routine pretreatment procedures to ensure adequate tissue permeability and preservation of nucleic acid targets, which are critical steps for successful hybridization efficiency [40].

A total volume of 10 µL of probe solution was applied to each slide, after which clean coverslips were carefully positioned to achieve even probe distribution while avoiding air bubble formation. Denaturation was carried out at 79 °C for 5 min to separate nucleic acid strands, followed by transfer of slides into a humidified chamber for hybridization at 37 °C for 24 h, enabling sequence-specific probe binding. Controlled temperature denaturation and prolonged hybridization are recognized as essential determinants of hybridization specificity and signal intensity in ISH-based assay.

After hybridization, coverslips were gently removed, and slides were immersed in saline–sodium citrate (SSC) wash buffer at 80 °C for 5 min to remove nonspecifically bound probe molecules. Sections were subsequently rinsed with distilled water and equilibrated in Tris-buffered saline (TBS). Detection of hybridized probes was achieved by applying 1–2 drops of Anti-Digoxigenin/Dinitrophenol (Anti-DIG/DNP) detection mixture to each slide, followed by incubation in a humid chamber at 37 °C for 15 min. Slides were then washed three times in freshly prepared TBS buffer for 1 min each to reduce background staining.

Chromogenic signal visualization was performed sequentially using enzyme-mediated color development. The alkaline phosphatase red (AP-Red) working solution was prepared by mixing 1 mL of AP-Red Solution B with one drop (30 µL) of AP-Red Solution A, and 1–2 drops were applied to each slide for 10 min at room temperature. During this incubation period, horseradish peroxidase green (HRP-Green) working solution was prepared by combining 1 mL of HRP-Green Solution B with two drops (2 × 20 µL) of HRP-Green Solution A. Following AP-Red development, slides were rinsed with distilled water for 2 min and subsequently incubated with HRP-Green solution for 10 min at room temperature. Dual-chromogen detection systems improve visualization of hybridization signals under brightfield microscopy and represent a commonly applied enhancement strategy in modern CISH protocols.

Slides were rinsed again with distilled water and counterstained with Nuclear Blue solution for 2 min to enable nuclear visualization. Specimens were then washed under cold running water, dehydrated in absolute ethanol, cleared in xylene, and mounted with coverslips. Hybridization signals were evaluated using brightfield light microscopy, which allows permanent signal preservation and morphological correlation, a key advantage of chromogenic in situ hybridization techniques compared with fluorescence-based methods [41].

Under normal conditions, two distinct chromogenic signals were expected within cell nuclei, corresponding to diploid gene copy number in cells observed during interphase or metaphase stages of the cell cycle, consistent with established interpretation criteria for chromogenic ICH analyze.

### 4.7. Evaluation of Factor Quantity

Slides prepared by IHC and CISH were examined with brightfield light microscopy. The semi-quantitative counting method was used to determine the relative frequency of protein-containing cells (for slides prepared with IHC) and the frequency of gene-signal-containing cells (for slides prepared with CISH). For each patient, five visual fields were randomly selected across the anterior–posterior extent of the tissue and analyzed. Microphotographs of slides were captured with a Leica DC 300F digital camera (Leica Microsystems Digital Imaging, Cambridge, UK). Afterwards, image processing was performed using Image Pro Plus 5.0 program (Media Cybernetics, Inc., Rockville, MD, USA). Evaluation of positively stained structures was performed according to identifiers summarized in Table 7 [41].

### 4.8. Statistical Analysis

All statistical analyses were performed using IBM SPSS software (Statistical Package for the Social Sciences), version 26.0 (IBM Corp., Chicago, IL, USA). A *p*-value of < 0.05 was considered statistically significant for all analyses. Because the semi-quantitative assessment of defense factor expression in tissue yielded ordinal, non-numeric data arranged in a fixed order, the analysis relied on descriptive and inferential statistics using non-parametric methods.

Median values were calculated for the semi-quantitative counts of protein-positive and gene-signal-positive cells. Differences between patient and control groups in the number of protein-containing or gene-signal-containing cells were evaluated using the Mann–Whitney U test and Spearman rank.

Spearman’s correlation coefficient (Rs) was used to calculate correlations between the evaluated proteins/genes with the following values: 0.0–0.2—very weak correlation, 0.2–0.4—weak correlation, 0.4–0.6—moderate correlation, 0.6–0.8—strong correlation, and 0.8–1.0—very strong correlation. Statistical analysis was performed with Statistical Product and Service Solutions (SPSS) Statistics version 29.0.0.0 (IBM Company, Chicago, IL, USA). Statistical significance of every calculation was determined with a *p*-value of < 0.05.

## 5. Conclusions

1. ***MYH3* protein presence was detected,** with significantly higher immunoreactivity in the muscle tissue of the unilateral right cleft lip suggesting a potential involvement of *MYH3* in altered muscle differentiation and structural organization associated with cleft-affected tissues.
2. **Significantly increased *IRF6* expression in epithelium** of cleft tissue compared with control samples indicates its crucial role in epithelial proliferation and differentiation.
3. **Gremlin showed limited distribution** in both patients and controls with the statistically significant difference in connective tissue suggesting a possible modulatory role of Gremlin in local tissue remodeling and signaling regulation in cleft-affected tissues.
4. **Significant positive correlations between *MYH3*, *IRF6*, and Gremlin expression** in cleft tissues indicate potential interactions between muscle differentiation pathways, epithelial regulatory mechanisms, and Gremlin–BMP signal pathways in the unilateral right cleft lip.

## Figures and Tables

**Table 1:** Semi-quantitative evaluation of *MYH3*, *GREM1*, *IRF6* immunoreactive cells in controls and cleft lip patients with IMH.

No.	MYH3				IRF6				Gremlin			
Patient	Ep	CT	End	M	Ep	CT	End	M	Ep	CT	End	M
1	+/++	0/+	0/+	+++/++++	++	0/+	+	0/+	0/+	0	0	0
2	0	+	+	+++/++++	+	0/+	0	0/+	0/+	0	0/+	0/+
3	+	0/+	+	++/+++	++	0/+	0	0/+	0/+	0	0	0/+
4	+	0/+	0/+	++/+++	+	0/+	0/+	0/+	00/+	0	0	0
5	+	0/+	0/+	+	0/+	0/+	0	0	0	0	0	0
6	+	0/+	0/+	0/+	++	0/+	+	0	+	0/+	0	0
7	++/+++	0/+	0/+	+++/++++	++/+++	0/+	0/+	0/+	0/+	0	0	0
8	+	0/+	0	+++/++++	0/+	0/+	0/+	0/+	0	0	0	0
9	++	+	0/+	++	+++	0/+	00/+	0/+	0/+	0	0	0/+
10	++	0/+	+/++	+/++	+/++	0/+	0	0	0	0	0	0
Median	+	0/+	0/+	++/+++	+/++	0/+	0/+	0/+	0/+	0	0	0
Control												
11	0	0	0	0	0	0/+	0	+	0	++/+++	0/+	+
12	+	0/+	+	++	0	0	0	0	0/+	0/+	0	0
13	0	0/+	0/+	0	0/+	0/+	0	0	+	+	0	0
14	+	0/+	0	0	0/+	0/+	0	0	0/+	0/+	0	0
15	+	0/+	0	0	0/+	0/+	0	0	0/+	0/+	0	0
16	+	0/+	0	0/+	0/+	0/+	0	0/+	0/+	0/+	0	0/+
Median	+	0/+	0	0	0/+	0/+	0	0	0/+	0/+	0	0

Abbreviations: MYH3—Myosin heavy chain 3; IRF6—interferon regulatory factor 6; Ep—epithelium; CT—connective tissue; End—endothelium; M—muscle tissue; 0—no gene-signal-containing cells in the visual field; 0/+—occasional occurrence of gene-signal-containing cells in the visual field; +—a few gene-signal-containing cells in the visual field; +/++—few to moderate gene-signal-containing cells in the visual field; ++—moderate gene-signal-containing cells in the visual field; ++/+++—moderate to numerous gene-signal-containing cells in the visual field; +++—numerous gene-signal- containing cells in the visual field. +++/++++—Numerous to abundant gene-signal-containing cells in the visual field.

**Table 2:** Statistically significant differences between the distributions of factor quantities in samples between factor-positive or gene-signal-containing cells and the control group using Mann–Whitney U test.

Patients	Control	Mann–Whitney U Test Value	Exact Sig. Value
Ep MYH3 IHC	Ep MYH3 IHC	15	0.118
CT MYH3 IHC	CT MYH3 IHC	20	0.313
End MYH3 IHC	End MYH3 IHC	24	0.093
M MYH3 IHC	M MYH3 IHC	4	0.003 *
Ep IRF6 IHC	Ep IRF6 IHC	4	0.003 *
CT IRF6 IHC	CT IRF6 IHC	25	0.635
End IRF6 IHC	End IRF6 IHC	12	0.056
M IRF6 IHC	M IRF6 IHC	22.5	0.428
Ep GREM1 IHC	Ep GREM1 IHC	25	0.635
CT GREM1 IHC	CT GREM1 IHC	2	<0.001 *
End GREM1 IHC	End GREM1 IHC	28	0.875
M GREM1 IHC	M GREM1 IHC	27.5	0.792
Ep IRF6 CISH	Ep IRF6 CISH	10.5	0.031 *
CT IRF6 CISH	CT IRF6 CISH	19.5	0.263
End IRF6 CISH	End IRF6 CISH	15	0.118
M IRF6 CISH	M IRF6 CISH	30	1.000
Ep GREM1 CISH	Ep GREM1 CISH	15	0.118
CT GREM1 CISH	CT GREM1 CISH	29	0.958
End GREM1 CISH	End GREM1 CISH	30	1.000
M GREM1 CISH	M GREM1 CISH	28.5	0.875

Abbreviations: MYH3—Myosin heavy chain 3; IRF6—interferon regulatory factor 6; GREM1—Gremlin 1; Ep—epithelium; CT—connective tissue; End—endothelium; M—muscle tissue; IHC—immunohistochemistry; CISH—chromogenic in situ hybridization; Mann–Whitney U—Mann–Whitney U test value. *—statistically significant value.

**Table 3:** Semi-quantitative evaluation of *IRF6* and *GREM1* gene-signal-containing cells in controls and cleft lip patients.

No.	IRF6				Gremlin			
Patient	Ep	CT	End	M	Ep	CT	End	M
1	+	0/+	0/+	0	0/+	0	0	0
2	++	0/+	0	0	0/+	0/+	0	0
3	++	0	0	0	0/+	0/+	0	0
4	+	+	0/+	0	0/+	0/+	0	0
5	0	0	0	0	0/+	0/+	0	++
6	++/+++	0/+	0/+	0	0/+	0/+	0	0
7	+/++	0/+	0	0	0/+	0/+	0	0
8	++	0/+	++	0	0/+	0	0	0
9	0/+	0/+	0/+	0	0/+	0	0	0
10	++/+++	0/+	0	0	0/+	0/+	0	0
Median	+/++	0/+	0/+	0	0/+	0/+	0	0
Control								
11	0	0/+	0	0	0	0/+	0	0
12	0	0	0	0	0	0	0	0
13	0	0	0	0	0	0	0	0
14	+	0/+	0	0	0/+	0/+	0	0
15	+	0	0	0	0/+	0/+	0	0/+
16	+	0/+	0	0	0/+	0/+	0	0
Median	0/+	0/+	0	0	0/+	0/+	0	0

Abbreviations: IRF6—interferon regulatory factor 6; Ep—epithelium; CT—connective tissue; End—endothelium; M—muscle tissue; 0—no gene-signal-containing cells in the visual field; 0/+—occasional occurrence of gene-signal-containing cells in the visual field; +—a few gene-signal-containing cells in the visual field; +/++—few to moderate gene-signal-containing cells in the visual field; ++—moderate gene-signal-containing cells in the visual field; ++/+++—moderate to numerous gene-signal-containing cells in the visual field.

**Table 4:** Statistically significant correlations between factor-positive and/or gene-signal-containing cells in the patient and control group.

Strength of Correlation	Correlation Between Factors	Rs	*p* < 0.005
Very strong positive (0.8–1.0)	IRF6 in M (IHC) and MYH3 in M (IHC) patient	0.826	0.003
Strong positive correlations (0.6–0.8)	IRF6 in End (IHC) and IRF6 in End (CISH) patient	0.764	0.013
	GREM1 in M (IHC) and MYH3 in CT (IHC) patient	0.764	0.010
	GREM1 in End (IHC) and MYH3 in CT (IHC) patients	0.667	0.035
Very strong negative (0.8–1.0)	GREM1 in CT (IHC) and MYH3 in Ep (IHC) control	−0.980	<0.001
	MYH3 in End (IHC) and GREM1 in CT (CISH)	−0.980	<0.001

Abbreviations: MYH3—Myosin heavy chain 3; IRF6—interferon regulatory factor 6; GREM1—Gremlin 1; Ep—epithelium; CT—connective tissue; End—endothelium; M—muscle tissue; IHC—immunohistochemistry; CISH—chromogenic in situ hybridization; Rs—Spearman’s Rho value; *p*—*p*-value.

**Table 5:** Description of the patients.

Patient Number	Age (Months)	Sex	Family Anamnesis
1	3	Male	
2	3	Male	
3	4	Female	
4	4	Male	Mother had hepatitis B. Grandmother from mother’s side had full cleft palate
5	4	Female	
6	4	Male	Mother had urinary tract infection
7	5	Male	
8	7	Male	
9	7	Female	Mother used gentamicin and ampicillin during pregnancy
10	18	Female	

**Table 6:** Description of controls.

Control Number	Age	Sex	Material Origin
1a	Newborn	Male	Birth asphyxia
4a	24 weeks old	Female	Abortion due to maternal indications
5a	Newborn	Female	Sudden infant death syndrome
1LSM	Newborn	Male	Operation material from frenectomy
2LSM	Newborn	Female	Operation material from frenectomy
5LSM	Newborn	Male	Operation material from frenectomy

**Table 7:** Scale used in the semi-quantitative evaluation.

Identifier Used	Explanation
0	No positive structures in the visual field (0%)
0/+	Occasionally positive structures (12.5%)
+	Few positive structures (25%)
+/++	Few to moderate number of positive structures (37.5%)
++	Moderate number of positive structures (50%)
++/+++	Moderate to numerous numbers of positive structures (62.5%)
+++	Numerous numbers of positive structures (75%)
+++/++++	Numerous to abundant number of positive structures (87.5%)
++++	Abundant number of positive structures (100%)

## Data Availability

The original contributions presented in this study are included in the article. Further inquiries can be directed to the corresponding author.

## Abbreviations

The following abbreviations are used in this manuscript:

MYH3	Myosin heavy chain 3
MYH8	Myosin heavy chain 8
MYH11	Myosin heavy chain 11
GREM1	Gremlin 1
IRF6	Interferon regulatory factor 6
IHC	Immunohistochemistry
CISH	Chromogenic in situ hybridization
RSU	Riga Stradins University
Ep	Epithelium
CT	Connective tissue
End	Endothelium
SPSS	Statistical Product and Service Solutions
U	Mann–Whitney U test value
TGF-β	Transforming growth factor beta
*p*	*p*-value
BMP	Bone morphogenetic protein
MEE	Medial edge epithelium
Rs	Spearman’s correlation coefficient

## References

[1] Dixon M.J., Marazita M.L., Beaty T.H., Murray J.C. (2011). Cleft lip and palate: Understanding genetic and environmental influences. Nat. Rev. Genet..

[2] Mossey P.A., Little J., Munger R.G., Dixon M.J., Shaw W.C. (2009). Cleft lip and palate. Lancet.

[3] Evangelista J.R., Bernardi C., Faria A.V., Bueno D.F. (2026). Matrix metalloproteinase-3 (MMP3) in non-syndromic cleft lip and palate: Extracellular matrix remodeling, developmental signaling, and molecular mechanisms. Biochem. Biophys. Res. Commun..

[4] Vaivads M., Balode E., Pilmane M. (2020). Factors affecting facial development and formation of cleft lip and palate: A literature review. Pap. Anthropol..

[5] Leslie E.J., Taub M.A., Liu H., Steinberg K.M., Koboldt D.C., Zhang Q., Carlson J.C., Hetmanski J.B., Wang H., Larson D.E. (2016). Genome-wide meta-analyses identify multiple loci for orofacial clefts. Nat. Commun..

[6] Jugessur A., Murray J.C. (2005). Orofacial clefting: Recent insights into a complex trait. Curr. Opin. Genet. Dev..

[7] Kondo S., Schutte B.C., Richardson R.J., Bjork B.C., Knight A.S., Watanabe Y., Howard E., Ferreira de Lima R.L., Daack-Hirsch S., Sander A. (2002). IRF6 mutations in Van der Woude syndrome and nonsyndromic clefts. Nat. Genet..

[8] Richardson R.J., Dixon J., Jiang R., Dixon M.J. (2009). Integration of IRF6 and Jagged2 signalling is essential for controlling palatal adhesion and fusion competence. Hum. Mol. Genet..

[9] Zucchero T.M., Cooper M.E., Maher B.S., Daack-Hirsch S., Nepomuceno B., Ribeiro L., Caprau D., Christensen K., Suzuki Y., Machida J. (2004). Interferon regulatory factor 6 (IRF6) gene variants and the risk of isolated cleft lip or palate. N. Engl. J. Med..

[10] Rahimov F., Jugessur A., Murray J.C. (2012). Genetics of nonsyndromic orofacial clefts. J. Dent. Res..

[11] Ingraham C.R., Kinoshita A., Kondo S., Yang B., Sajan S., Trout K., Malik M., Goudy S., Lovett M., Murray J.C. (2006). Abnormal skin, limb and craniofacial morphogenesis in mice deficient for interferon regulatory factor 6 (Irf6). Nat. Genet..

[12] Smane L., Pilmane M. (2015). IRF6, RYK, and PAX9 expression in facial tissue of children with cleft palate. Int. J. Morphol..

[13] Krivicka-Uzkurele B., Pilmane M., Skagers A., Erts R. (2016). Expression of interferon regulatory factor 6, muscle segment homeobox 1, paired box 9, homeobox B3, and related tyrosine kinases in cleft-affected cartilage. J. Oral Maxillofac. Surg..

[14] Zuniga E., Rippen M., Alexander C., Schilling T.F., Crump J.G. (2011). Gremlin regulates distinct roles of BMP and Endothelin 1 signaling in dorsoventral patterning of the facial skeleton. Development.

[15] Michos O., Panman L., Vintersten K., Beier K., Zeller R., Zuniga A. (2004). Gremlin-mediated BMP antagonism induces the epithelial-mesenchymal feedback loop during limb bud outgrowth. Dev. Biol..

[16] Ludwig K.U., Mangold E., Herms S., Nowak S., Reutter H., Paul A., Becker J., Herberz R., AlChawa T., Nasser E. (2012). Genome-wide meta-analyses of nonsyndromic cleft lip with or without cleft palate identify six new risk loci. Nat. Genet..

[17] Mangold E., Ludwig K.U., Birnbaum S., Baluarda I., Ferrian M., Herms S., Reutter H., de Assis N.A., Chawa T.A., Schuenke H. (2010). Genome-wide association study identifies two susceptibility loci for nonsyndromic orofacial clefts. Nat. Genet..

[18] Li B., Kuriyama S., Moreno M., Mayor R., Selleck M.A., Bronner M.E. (2013). Gremlin 1 is required for the de-velopment of the cranial base and the tongue. Development.

[19] Mostowska A., Hozyasz K.K., Wójcicki P., Żukowski K., Dąbrowska A., Lasota A., Zadurska M., Radomska A., Dunin-Wilczyńska I., Jagodziński P.P. (2015). Association between polymorphisms at the GREM1 locus and the risk of nonsyndromic cleft lip with or without cleft palate in the Polish population. Birth Defects Res. A Clin. Mol. Teratol..

[20] Rafighdoost H., Poudineh A., Bahari G., Ghaffari H., Hashemi M. (2020). Association of genetic polymorphisms of GREM1 gene with susceptibility to non-syndromic cleft lip with or without cleft palate in an Iranian population. Fetal Pediatr. Pathol..

[21] Toydemir R.M., Rutherford A., Whitby F.G., Jorde L.B., Carey J.C., Bamshad M.J. (2006). Mutations in muta-tions in MYH3 cause Freeman-Sheldon syndrome and Sheldon-Hall syndrome. Nat. Genet..

[22] Shih H.P., Gross M.K., Kioussi C., McLoon L.K., Andrade F.H. (2013). Craniofacial musculature development. Craniofacial Muscles: A New Framework for Understanding the Effector Side of Craniofacial Muscle Control.

[23] Zieba J., Zhang W., Chong J., Forlenza K.N., Martin J.H., Heard K., Grange D.K., Butler M.G., Kleefstra T., Lachman R.S. (2017). A postnatal role for embryonic myosin revealed by MYH3 mutations that alter TGFβ signaling and cause autosomal dominant spondylocarpotarsal synostosis. Sci. Rep..

[24] Vaiman A., Fritz S., Beauvallet C., Boussaha M., Grohs C., Daniel-Carlier N., Relun A., Boichard D., Vilotte J.L., Duchesne A. (2022). Mutation of the MYH3 gene causes recessive cleft palate in Limousine cattle. Genet. Sel. Evol..

[25] Huang Z., Zhang C., Sun M., Ma A., Chen L., Jiang W., Xu M., Bai X., Zhou J., Zhang W. (2024). Proteomic analysis illustrates the potential involvement of motor proteins in cleft palate development. Sci. Rep..

[26] Park J.W., McIntosh I., Hetmanski J.B., Jabs E.W., der Van Kolk C.A., Wu-Chou Y.-H., Chen P.K., Chong S.S., Yeow V., Jee S.H. (2007). Association between IRF6 and nonsyndromic cleft lip with or without cleft palate in four populations. Genet. Med..

[27] Moretti F., Marinari B., Lo Iacono N., Botti E., Giunta A., Spallone G., Garaffo G., Vernersson-Lindahl E., Merlo G., Mills A.A. (2010). A regulatory feedback loop involving p63 and IRF6 links the pathogenesis of two genetically different human ectodermal dysplasias. J. Clin. Investig..

[28] Ke C.Y., Xiao W.L., Chen C.M., Lo L.-J., Wong F.-H. (2015). IRF6 is the mediator of TGFβ3 during regulation of the epithelial mesenchymal transition and palatal fusion. Sci. Rep..

[29] Nasroen S.L., Maskoen A.M., Soedjana H., Hilmanto D., Gani B.A. (2022). IRF6 rs2235371 as a risk factor for non-syndromic cleft palate only among the Deutero-Malay race in Indonesia and its effect on the IRF6 mRNA expression level. Dent. Med. Probl..

[30] Chen D., Zhao M., Mundy G.R. (2004). Bone morphogenetic proteins. Growth Factors.

[31] Nie X., Luukko K., Kettunen P. (2006). BMP signalling in craniofacial development. Int. J. Dev. Biol..

[32] Mishina Y., Snider T.N. (2014). Neural crest cell signaling pathways critical to craniofacial development and disease. Birth Defects Res. C Embryo Today.

[33] Zuniga A., Laurent F., Lopez-Rios J., Klasen C., Matt N., Zeller R. (2012). Conserved cis-regulatory regions in a large genomic landscape control SHH and BMP-regulated Gremlin1 expression in mouse limb buds. BMC Dev. Biol..

[34] Lan Y., Jia S., Jiang R. (2015). Molecular patterning of the mammalian dentition. Semin. Cell Dev. Biol..

[35] Graf D., Malik Z., Hayano S., Mishina Y. (2016). Common mechanisms in craniofacial anomalies and skeletal patterning. Semin. Cell Dev. Biol..

[36] Ramos-Vara J.A. (2005). Technical aspects of immunohistochemistry. Vet. Pathol..

[37] Shi S.R., Shi Y., Taylor C.R. (2011). Antigen retrieval immunohistochemistry: Review and future prospects. J. Histochem. Cytochem..

[38] Buchwalow I.B., Böcker W. (2010). Immunohistochemistry: Basics and Methods.

[39] Reynolds K., Kumari P., Sepulveda E., Rincon-Limas D., Kondoh H. (2019). Epithelial–mesenchymal signaling in craniofacial development. Front. Physiol..

[40] Roche Diagnostics (2016). INFORM^®^ CISH DNA Probe and Detection Kit: Technical Guide.

[41] Rehman Z.U., Ahmad Fauzi M.F., Wan Ahmad W.S.H.M., Abas F.S., Cheah P.L., Chiew S.F., Looi L.-M. (2024). Review of in situ hybridization stain images using computational techniques. Diagnostics.

